# Application of radiosurgical techniques to produce a primate model of brain lesions

**DOI:** 10.3389/fnsys.2015.00067

**Published:** 2015-04-24

**Authors:** Jun Kunimatsu, Naoki Miyamoto, Masayori Ishikawa, Hiroki Shirato, Masaki Tanaka

**Affiliations:** ^1^Systems Neuroscience Laboratory, Department of Physiology, Hokkaido University School of MedicineSapporo, Japan; ^2^Department of Medical Physics, Hokkaido University School of MedicineSapporo, Japan; ^3^Department of Radiation Oncology, Hokkaido University School of MedicineSapporo, Japan

**Keywords:** radiation, LINAC, frontal eye field, saccade, smooth pursuit, monkey

## Abstract

Behavioral analysis of subjects with discrete brain lesions provides important information about the mechanisms of various brain functions. However, it is generally difficult to experimentally produce discrete lesions in deep brain structures. Here we show that a radiosurgical technique, which is used as an alternative treatment for brain tumors and vascular malformations, is applicable to create non-invasive lesions in experimental animals for the research in systems neuroscience. We delivered highly focused radiation (130–150 Gy at ISO center) to the frontal eye field (FEF) of macaque monkeys using a clinical linear accelerator (LINAC). The effects of irradiation were assessed by analyzing oculomotor performance along with magnetic resonance (MR) images before and up to 8 months following irradiation. In parallel with tissue edema indicated by MR images, deficits in saccadic and smooth pursuit eye movements were observed during several days following irradiation. Although initial signs of oculomotor deficits disappeared within a month, damage to the tissue and impaired eye movements gradually developed during the course of the subsequent 6 months. Postmortem histological examinations showed necrosis and hemorrhages within a large area of the white matter and, to a lesser extent, in the adjacent gray matter, which was centered at the irradiated target. These results indicated that the LINAC system was useful for making brain lesions in experimental animals, while the suitable radiation parameters to generate more focused lesions need to be further explored. We propose the use of a radiosurgical technique for establishing animal models of brain lesions, and discuss the possible uses of this technique for functional neurosurgical treatments in humans.

## Introduction

The causal roles of specific brain areas in particular functions have been elucidated by studying the effects of lesions on behavior. For example, neuropsychological studies on patients with hippocampal damage helped to establish the fundamental principles in our understanding of how memory functions are organized (Penfield and Milner, 1958; see for review, Squire, 2009), and those in subjects with lesions in the cerebral cortex revealed that higher cognitive functions, such as thought, motivation, and social behavior, are relevant to association areas in the frontal cortex (Harlow, 1848; Damasio et al., 1994). Further, lesion studies using monkeys have also characterized the functional roles of various cortical and subcortical regions, including the prefrontal cortex (Passingham, 1975), premotor cortex (Moll and Kuypers, 1977), inferior temporal cortex (Bachevalier and Mishkin, 1994), and amygdala (Klüver and Bucy, 1997). More recent studies have used a variety of techniques to create localized brain lesions, such as ligation of the feeding artery (Gonzalez et al., 2004), cooling (Lomber and Malhotra, 2008; Peel et al., 2014), electric coagulation (Majkutewicz et al., 2010), injection of neurotoxic (Rudebeck et al., 2013) or neuroactive (Hikosaka and Wurtz, 1985; Grabli et al., 2004) agents, and repetitive magnetic stimulation (Muellbacher et al., 2002). Nevertheless, neurosurgery remains advantageous for creating relatively large, circumscribed brain lesions in experimental animals (Mansouri et al., 2007; Takaura et al., 2011) and to examine the processes of functional recovery (Nishimura et al., 2007; Murata et al., 2008; Darling et al., 2009). However, neurosurgery is a highly invasive procedure requiring a craniotomy, and it is technically impossible to make subcortical lesions without damage to the cerebral cortex.

Nowadays, radiosurgery has become a well-established treatment alternative for various neurosurgical diseases, including those with indications of functional neurosurgery (Niranjan and Lunsford, 2000). Radiosurgery using a linear accelerator (LINAC) is designed to deliver a high dose of focused radiation to a specific target to elicit local radiobiological responses. This is a simple, non-invasive technique with no need of craniotomy and can be performed in a short period of time. The radiobiological response depends on the radiation dose, the nature of the tissue, and the time elapsed after irradiation. The radiation effects are found in neurons later than in glial cells or vascular endothelial cells, because neurons are basically postmitotic and do not frequently divide (Greene-Schloesser et al., 2012). In this study, we attempted to adopt a radiosurgical technique for potential application in systems neuroscience. We developed methods to deliver radiation to anesthetized macaque monkeys, and examined the time course of behavioral changes and the development of lesions over months. Specifically, we chose the frontal eye field (FEF) as the irradiation target because of its measurable motor output (i.e., eye movements) and relatively well-understood neural circuitry. Our data show that radiosurgical techniques are useful for establishing animal models of brain lesions.

## Methods

### Animal Preparation

Three Japanese monkeys (*Macaca fuscata*; age range, 4–11 years; weight, 5–7 kg) were used. All experimental protocols were approved by the Hokkaido University Animal Care and Use Committee. The procedures of animal preparation were identical to those previously described (Kunimatsu and Tanaka, 2010). Initially, monkeys were habituated to sit in a primate chair. The animals were then implanted with a pair of head holders and an eye coil, using sterile procedures under general isoflurane anesthesia. Analgesics were administered during each surgery and over the following few days. After full recovery from surgery, the monkeys were trained on oculomotor tasks. During the training and subsequent experimental sessions, the animal’s head was secured to the primate chair in a darkened booth, and horizontal and vertical eye position signals were recorded using the search coil technique (MEL-25; Enzanshi Kogyo, Chiba, Japan). Water intake of monkeys was controlled on a daily basis so that they were motivated to perform the tasks.

### Visual Stimuli and Behavioral Tasks

Experiments were controlled using a Windows-based real-time data acquisition system (TEMPO; Reflective Computing, St Louis, MO, USA). Visual stimuli were presented on a 24-inch cathode-ray tube monitor (refresh rate, 60 Hz) positioned 38 cm away from the eyes, and subtended at a visual angle of 64 × 44°. We used three oculomotor paradigms: visually-guided saccade task, memory-guided saccade task, and step-ramp smooth pursuit task. In the visually-guided saccade task, a saccade target appeared at the time of the fixation point (FP) offset, and monkeys made a saccade within 800 ms. In the memory-guided saccade task (Hikosaka and Wurtz, 1983), a visual cue was presented briefly (200 ms) during central fixation, and the monkeys were required to remember its location and maintain fixation for an additional delay interval (800 ms). Within 800 ms of the FP offset, the monkeys made a saccade to the cue location to obtain a liquid reward. Although the animals were allowed to respond with a relatively long reaction time, they made saccades within 400 ms in most trials except for several months after irradiation (Figure 4C). In both saccade tasks, the target was presented 16° eccentrically in one of six predetermined directions (60° apart, starting with the right direction). In the step-ramp pursuit task (Rashbass, 1961), the FP was extinguished after a random 800–1200 ms interval, and a white moving target appeared 1.4–2.8° from the location of the initial fixation. The target moved toward the initial fixation location at 10, 15, or 20°/s, such that it crossed the fixation location 140 ms after motion onset. After an excursion for 1140–2140 ms, the target stopped and remained visible for an additional 900–1300 ms. Monkeys were required to move their eyes within a 2–4° window surrounding the target location throughout the trial, except for the initial 300-ms interval of target motion. These tasks were presented in three different blocks in monkeys *N* and *R*. In monkey *C*, all trials were presented in a pseudorandom order within a block that consisted of 36 different trials.

### Radiosurgical Procedures

**Figure 1:**
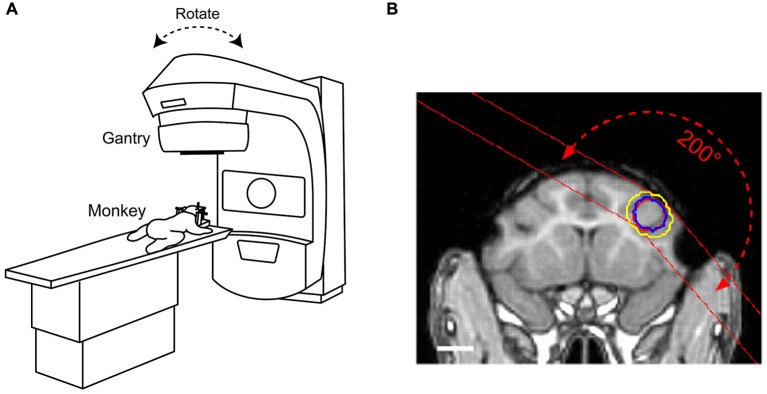
**Radiosurgical procedures to ablate macaque brain using a linear accelerator (LINAC). (A)** Under deep anesthesia, monkeys were placed on a moveable table with their heads fixed to the stereotaxic instrument. The gantry rotated around the predefined isocenter located in the target volume. **(B)** Location of the radiation target overlaid on a T1-weighted magnetic resonance (MR) image for monkey *C*. Red circle placed at the right frontal cortex indicates target volume (diameter 10 mm). The dose was delivered using multiple arc irradiation methods. Blue and yellow circles indicate 80 and 60% isodose lines, respectively. Red dashed line with arrows illustrates the range of X-ray paths through the arc irradiation. White bar indicates 10 mm.

**Figure 2:**
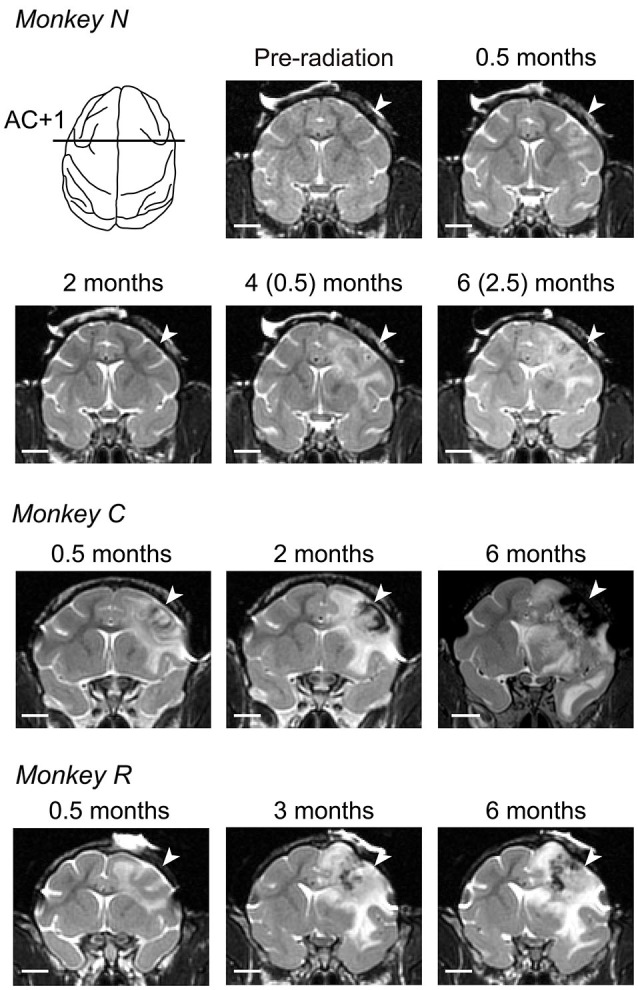
**Development of radiosurgical lesions**. T2-weighted coronal images at the level of 1 mm anterior to the anterior commissure (AC) are compared across different times from irradiation for three monkeys. For monkey *N*, we delivered the second irradiation at 99 days after initial irradiation, and the numbers in parentheses indicate months after second irradiation. White arrow on each panel denotes location of irradiation. White bar indicates 10 mm.

We initially collected three-dimensional images of a specimen of macaque skull with an MRI-compatible stereotaxic frame (Narishige, Tokyo) using computed tomography (CT, GE, Optima CT580) and MRI (GE, Signa 1.5T). We then collected MAGNETIC RESONANCE (MR) images of monkeys fixed with the same stereotaxic frame, and determined the target region of radiosurgery that was centered at the genu of the right arcuate sulcus. According to the CT/MR fusion images of the stereotaxic frame, the planning of irradiation was performed with a radiation treatment planning system (Phillips, Pinnacle^3^ ver. 9.0). Figure 1A illustrates the radiosurgical procedure using the LINAC system (Varian Medical Systems, Clinac 6EX). In this system, the gantry can rotate 360° around the predefined isocenter with the mechanical center located within a 1.0-mm radius. During irradiation, monkeys were anesthetized with sodium pentobarbital (20–25 mg/kg, i.p.), administered analgesics (pentazocine, 0.25 mg/kg, i.m.), and fixed to stereotaxic frames (Narishige, Tokyo, Japan). The prescribed dose was defined at ISO center, and the arc irradiation method was applied to concentrate a radiation dose covering the area of the anterior bank of the accurate sulcus. The target area (field size: 10 × 8 mm) included both gray matter and adjacent white matter (Figure 1B, monkey *R*, 150 Gy; monkey *N*, 130–150 Gy; monkey *C*, 135 Gy). The dose was selected based on previous studies in rats that used 25–200 Gy (Kondziolka et al., 1992; Ishikawa et al., 1999). The gantry angle for the arc irradiation ranged from 140–300° (rotated clockwise through 0–360° position). We used MR imaging (GE, Signa 1.5T or Siemens, Prisma 3T) to follow development of the radiosurgical lesions (T2-weighted images, TR 4900 ms, TE 80.6 ms, 1.5-mm-thick slices or TR 3600 ms, TE 259 ms, 0.5-mm-thick slices). Because the first monkey (*N*) did not exhibit any obvious lesions or behavioral changes until the second month following the first radiosurgery (Figures 2, 4C), we irradiated the same area again for this particular monkey.

### Histological Procedures

At the end of the experiments, animals were deeply anesthetized with sodium pentobarbital (>50 mg/kg, i.p.) and perfused with 0.1 M phosphate buffer followed by 3.5% formalin. The brain was blocked and equilibrated with 30% sucrose, and was cut into coronal sections at 100 μm thicknesses using a freezing microtome. Each section was stained with cresyl violet or hematoxylin and eosin (H&E).

### Data Acquisition and Analyses

Horizontal and vertical eye position signals were obtained directly from the eye coil system. The eye movement data were digitized and sampled at 1 kHz during the experiments, and were stored in files for subsequent off-line analyses that were performed using Matlab (Mathworks, Natick, MA, USA). We collected the data from at least 50 trials for each behavioral task in each daily session, and the sessions were repeated 2–5 times per week. For the visually-guided saccade task and the memory-guided saccade task, data were included for further analysis if saccades were generated within 800 ms following the FP offset, and if the endpoint of the saccade was within 5° of the cue location (8° for monkey *N*). Saccade latency was defined as the time from FP offset to the time of saccade initiation. Saccade accuracy was quantified by measuring the distance between the saccade endpoint and the location of the visual stimulus. For memory-guided saccades, the proportion of error trials with an early saccade toward the cue during the delay period was separately computed to quantify the effects of irradiation (Figure 4C, bottom row). For smooth pursuit eye movements, eye velocities during the initiation (100–200 ms and 150–250 ms after target motion onset for monkeys *R* and *C*, respectively) and the maintenance (300–500 ms and 350–550 ms) of pursuit were separately measured. Because monkey *N* was unable to generate a high-gain smooth pursuit, even before irradiation possibly due to the preceding physiological experiments on the thalamus (Kunimatsu and Tanaka, 2010) and the medial frontal cortex (Kunimatsu and Tanaka, 2012), the data from this monkey was excluded from analyses of smooth pursuit eye movements.

## Results

### Development of Radiosurgical Lesions

We irradiated the right FEF in three macaque monkeys (130–150 Gy, Figure 1B). Consistent with previous experiments in cats (Blatt et al., 1994), these doses were sufficient to induce localized brain lesions in monkeys. The effects of radiation in the target area were assessed with the aid of MR imaging performed almost every month after radiosurgery. The T2-weighted MR images in Figure 2 show the evolution of the lesion over 6 months. The images taken at 0.5 months (12, 11, and 20 days for monkeys *N*, *C*, and *R*, respectively) following irradiation demonstrated characteristics of brain edema in the white matter. For monkey *N*, we irradiated again with 150 Gy at 99 days after the initial irradiation session, because tissue edema in this animal decreased progressively and completely dissipated within 2 months (74 days). For monkeys *C* and *R*, brain edemas extended widely into the white matter and invaded the right external and internal capsules several months after irradiation. Further, the MR images also showed hemorrhage around the core of the irradiation target (Figure 2, low intensity areas). The mass effects with midline shift toward the contralateral hemisphere was found in images at 2 months (56 days) in monkey *C* and 3 months (82 days) in monkey *R*. These lesions and hemorrhages developed during the course of 6 months following irradiation (173, 187, and 187 days for monkeys *N, C*, and* R*, respectively). In addition to the large area in the white matter, the surrounding gray matter was also damaged.

**Figure 3:**
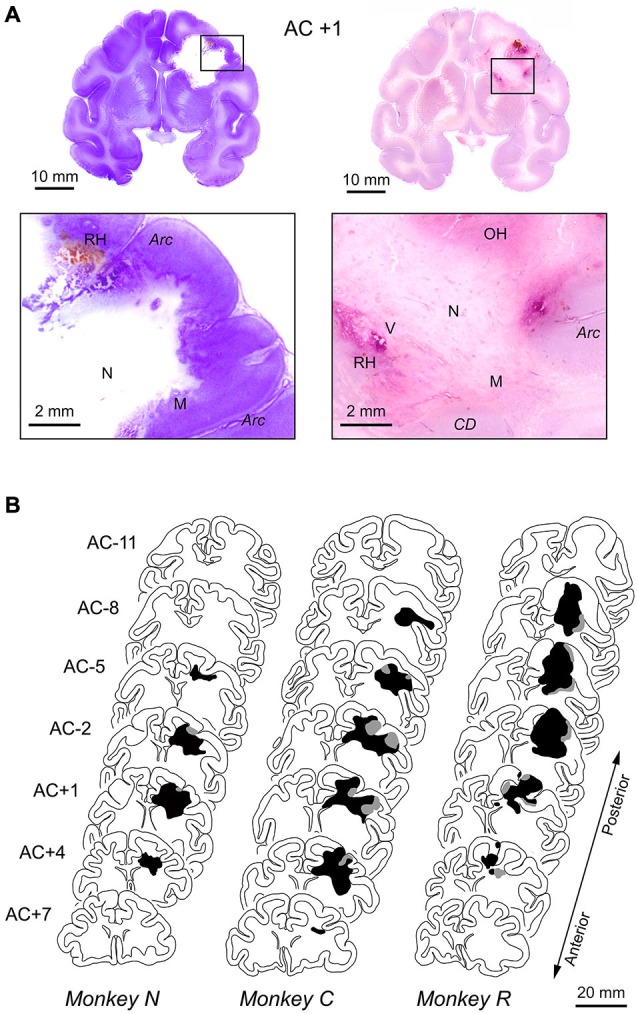
**Histological reconstruction of radiosurgical lesions. (A)** Representative histological sections stained with cresyl violet (left panels) or H&E (right panels). Coronal sections at the level of 1 mm anterior to the anterior commissure (AC) in monkey *N*. The brain was removed 6 months following the initial 130-Gy irradiation (2.5 months after the second 150-Gy irradiation). The necrotic lesion (N) was primarily in the white matter, and the surrounding tissue was composed of microglial cells (M). Some recent and old hemorrhages (RH, OH) were found in areas of hypervascularity (V). *CD*, caudate nucleus; *Arc*, arcuate sulcus. **(B)** Extent of radiosurgical lesions. Black and gray areas indicate locations of necrotic lesion and hemorrhages observed on histological sections, respectively. The levels of frontal sections are shown as the position relative to AC.

At the end of the 6-month observation period, we obtained histological sections from all three monkeys and performed pathological verification of radiosurgical lesions. The representative sections from monkey *N* shown in Figure 3A demonstrated that the radiosurgical lesion extended ventrally to the striatum and medially to the cingulate cortex. The necrotic tissues accompanying neuronal calcifications were not stained with the cresyl violet (lower left panel). The increment of cell density consisted of rod shaped microglia and astrocyte was observed at the border between normal and necrotic tissues. Gray matter surrounding the lesion was clearly necrotic, but seemingly to a lesser extent than the white matter (lower left panel). Vascular proliferations and glial reactions in the necrotic cavity were also identified on the histological sections stained with H&E. Some recent and old hemorrhages were found in and around the lesion, and these hemorrhages were accompanied by vascular proliferation in the hypervascular region (lower right panel). Thus, the radiation lesions that we observed in the MR images constituted both hemorrhages and necrotic tissue, consistent with previous results (Blatt et al., 1994).

Figure 3B illustrates the antero-posterior extent of irradiated lesions consisted of hemorrhages (gray area) and necrotic tissue (black area), which were identified on histological sections. For all animals, the radiosurgical lesions were centered at the genu of the arcuate sulcus (labeled AC +1 or AC –2) and extended anteriorly to the prefrontal cortex and posteriorly to the premotor cortex. Following radiosurgery, pathological changes seemed to steadily develop over months, while the first monkey (*N*) showed some improvement on MRI at 2 months (Figure 2). This could be partly because the irradiation dose for this animal was slightly less than for the others (130 vs. 135–150 Gy). Although the long-term necrotic changes might also occur following the first irradiation in this monkey, we were unable to examine this, because the monkey received a second irradiation after the initial recovery (Figure 2).

### Effects of Radiosurgical FEF Lesions on Saccades

**Figure 4:**
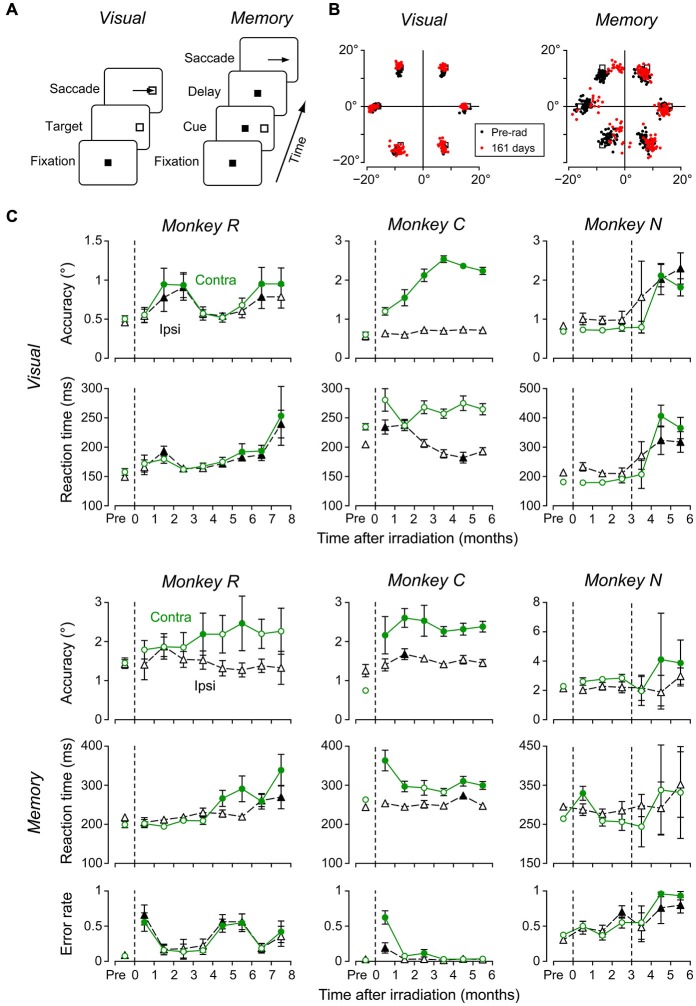
**Effects of radiosurgical frontal eye field (FEF) lesions on saccades. (A)** Sequence of events in the two saccade tasks. In the visually-guided saccade task, monkeys made an immediate saccade toward a visible target (left), while in the memory-guided saccade task, they made a saccade to the location of a previously flashed target (right). Color of the fixation point (FP) differed between tasks. **(B)** Distributions of saccade end points obtained before and at 161 days following irradiation. **(C)** Data of visually guided (top two rows) and memory-guided (bottom 3 rows) saccades in three monkeys. Each data point plots the mean (±95% confidence interval) of 8–22 daily sessions. Dashed vertical lines indicate days of radiosurgery. On each panel, circles and triangles denote contraversive and ipsiversive saccades, respectively. Filled symbols represent data differing from those before irradiation (multiple comparisons with Scheffé’s method, *p* < 0.05).

To examine the effects of irradiation on brain functions, we compared eye movements before and following radiosurgery in the conventional saccade tasks (Figure 4A, Methods). Figure 4B plots the sample distributions of saccade endpoints in the visually-guided saccade trials (left) and the memory-guided saccade trials (right) in monkey *R*. The endpoint error of visually-guided saccades measured on 161 days following irradiation (red dots) was slightly, but statistically, greater than at 45 days before irradiation (black dots, unpaired *t*-test, *p* < 0.05). More pronounced irradiation effects were found for contraversive (leftward) memory-guided saccades on the same day (unpaired *t*-test, *p* < 0.01).

To assess the time course of irradiation effects, we measured eye movements 2–5 days a week during 6–8 months following radiosurgery. Figure 4C plots saccade accuracy and reaction time for each month in three monkeys (top four rows), as well as the proportion of error trials with early saccades toward the cue in the memory-guided saccade task (bottom row). Each data point indicates the mean (±95% confidence intervals) of 8–22 daily sessions in each month. Filled symbols denote data that exhibited a significant difference from those obtained before irradiation (multiple comparisons with Scheffé test, *p* < 0.05). Although the timing of behavioral changes following irradiation varied between monkeys, we found some consistent effects. In accordance with the appearance of the brain edema shortly after irradiation, the performance of both visually-guided and memory-guided saccades became worse (after the second irradiation for monkey *N*), and the proportions of erroneous memory-guided saccades increased after a few months. Interestingly, these changes recovered within several months in monkeys *R* and *C* (accuracy and reaction time of visually-guided saccades, error rate during the memory-guided saccade task). Although all three monkeys consistently exhibited impaired contraversive saccades, the irradiation effects were also found in some parameters of ipsiversive saccades in 2 monkeys (*R* and *N*). Besides the impairment of eye movements, we also found subtle motor deficits in the contralateral limb approximately 2 months following irradiation in two monkeys (*R* and *C*). Unfortunately, we were unable to quantitatively analyze these deficits in the present study.

### Effects of Radiosurgical Lesions on Smooth Pursuit

**Figure 5:**
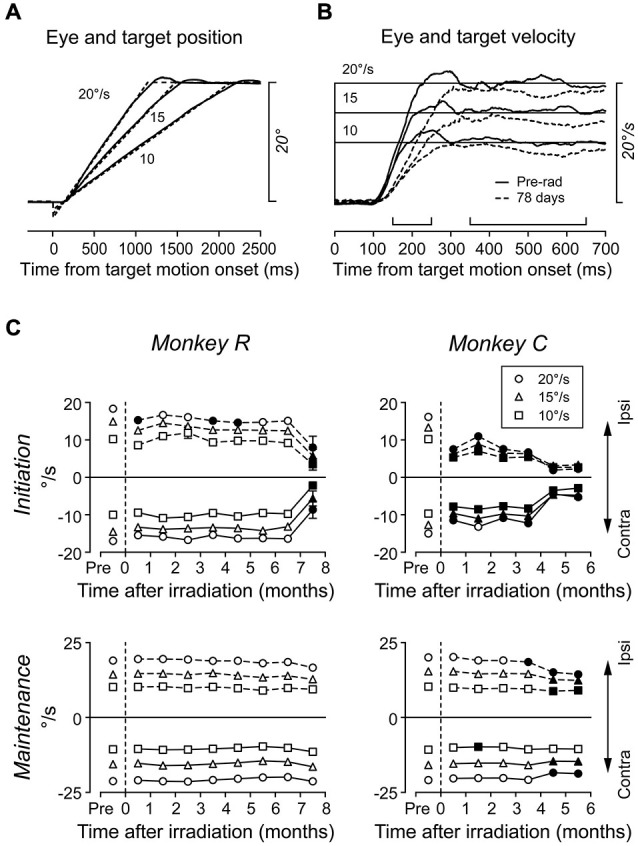
**Effects of radiosurgical FEF lesions on smooth pursuit. (A)** Series of target trajectories and examples of eye positions. The target moved toward the fixation location at 10, 15, or 20°/s so that it crossed the fixation location 140 ms after motion onset. **(B)** Averages of desaccaded eye velocity for rightward (ipsilateral to the irradiation sites) target motion before (solid trace) and at 78 days following (broken trace) irradiation. Upward brackets indicate times of pursuit initiation (150–250 ms) and maintenance (350–650 ms). **(C)** Eye velocity during pursuit initiation and maintenance. Each data point plots the mean of 10–22 daily sessions. Different symbols indicate data for different target speeds. Filled symbols indicate data differing from those before irradiation (Scheffé, *p* < 0.05).

Because the caudal region of the FEF is implicated in smooth pursuit eye movements (Gottlieb et al., 1993; Tanaka and Fukushima, 1998; Tanaka and Lisberger, 2001), we also examined the effects of radiosurgery on smooth pursuit. Monkeys were trained to track a target moving at 10, 15, or 20°/s along the horizontal meridian (Figure 5A). Figure 5B compares the average traces of desaccaded eye velocity during rightward (ipsilateral to irradiation) pursuit in daily sessions before (solid trace) and at 78 days following irradiation (broken trace) in monkey *C*. Eye velocity during pursuit initiation exhibited lower acceleration following irradiation, resulting in a smaller mean value (10°/s target motion, 9.6 vs. 5.4°/s; 15°/s, 12.4 vs. 6.1°/s; 20°/s, 16.8 vs. 8.5°/s; unpaired *t*-test, *p* < 0.01 for all target speeds). Further, eye velocity during pursuit maintenance also showed a significant reduction (10°/s, 10.1 vs. 8.5°/s; 15°/s, 15.4 vs. 13.3°/s; 20°/s, 19.9 vs. 18.1°/s; unpaired *t*-test, *p* < 0.01). These results were consistent with a previous study showing that inactivation of the FEF reduced pursuit gain both during initiation and maintenance of pursuit (Shi et al., 1998).

Figure 5C illustrates the time course of pursuit deficits over many months. As previously described, each data point indicates the mean of daily sessions in each month. For monkey *R*, deficits in ipsiversive pursuit initiation were only occasionally observed until 7 months, while impaired pursuit initiation in both directions became obvious by 8 months. For monkey *C*, eye velocity during pursuit initiation greatly decreased just after irradiation and worsened progressively during the following 6 months. In contrast, the gain of pursuit maintenance was generally stable for both monkeys, and it only reduced after several months in monkey *C*. These results show that pursuit initiation was a more sensitive measure to probe the functional deficits in the FEF than pursuit maintenance.

## Discussion

To the best of our knowledge, this is the first study to perform a long-term follow-up of the effects of localized irradiation on brain functions and MR images in non-human primates. The initial signs of cytotoxic edema were found soon after irradiation. Based on MR images, edema and necrotic lesions gradually and steadily extended over 6 months. In contrast, the impairments of oculomotor performance appeared over several weeks following irradiation, but somewhat recovered over the course of several months (Figure 4C, monkeys *R* and *C*). A few months following recovery, the deficits in eye movements reappeared and gradually progressed until the end of our observation period. Thus, during the early stages of radiosurgical treatment, the functional deficits were dissociated from the abnormal findings in MRI. These results provide novel information on the functional and pathological changes after localized irradiation. The radiosurgical technique would be useful for the research in systems neuroscience involving establishing animal models of brain lesions.

### Characteristics of Radiosurgical Lesions

Radiosurgery is an important option for the treatment of brain tumors and vascular malformations. Localized irradiation is effective for these diseases, because tumor cells and vascular endothelial cells rapidly divide. However, radiation is generally ineffective for postmitotic neurons (Li et al., 1996; Belka et al., 2001). Nevertheless, even low-dose irradiation to the brain is known to cause cognitive impairments over the course of years (Greene-Schloesser et al., 2012). Furthermore, radiosurgical treatment has recently been applied to functional neurosurgery; localized irradiation is expected to generate focal irreversible lesions in normal brain tissues in patients with obsessive compulsive disorder (Kondziolka et al., 2011) or Parkinson’s disease (Ohye et al., 2012). To make this technique fully available for inducing therapeutic brain lesions in patients, as well as in experimental animals, the influence of localized irradiation on neurons and surrounding glial and vascular endothelial cells need to be further clarified.

Although several studies examined the relationship between cellular loss and brain functions in rats, they conducted almost whole-brain irradiation and studied the effects over a short period of time (Maesawa et al., 2000; Liscák et al., 2002). In the present study, we delivered localized radiation in monkeys and examined specific motor functions for several months. We found that irradiation affected normal brain tissues through local hemorrhage and necrosis following long-lasting edema. The first sign of irradiation effects was widespread edema in the white matter during the month following irradiation. Hemorrhages and necrosis followed the edema, and some recent and old hemorrhages were found around the postnecrotic cavity in histological sections.

The extent of necrotic lesions was in good agreement with a previous study showing that a dose of 150 Gy induced circumscribed lesions measuring 8–13 mm in diameter at 6–24 weeks following irradiation in vervet monkeys (De Salles et al., 2001). Other studies also show that the white matter is more sensitive to radiation than the gray matter, likely because white matter contains many vascular endothelial cells (Schultheiss and Stephens, 1992; Greene-Schloesser et al., 2012). It has been well established that irradiation of capillary beds precedes necrosis of surrounding tissues (Kamiryo et al., 2001). Thus, vascular damage in the white matter might be an important cause of brain lesions following radiosurgery.

### Relationship Between Radiosurgical Lesions and Oculomotor Performance

In this study, we chose the FEF as a target of radiosurgical lesions. Because the impairments following surgical ablation of the FEF are compensated by other oculomotor structures (Schiller et al., 1979), one might argue that the radiation effects were more than what we observed in this study. However, we found severe oculomotor deficits following irradiation and were able to quantitatively evaluate the time course of impairments throughout our observation period. The reproducibility of the radiosurgical effects, especially the transient functional recovery from the acute state, need to be examined in other systems in future studies.

Following unilateral irradiation to the FEF, all monkeys consistently showed contraversive saccade deficits, while impairments of some aspects of ipsiversive saccades were also found in two out of three monkeys (Figure 4C, left two columns). The directional effects of irradiation indicated that the changes in oculomotor behavior were mostly attributed to the local lesion in the FEF, but not to changes in general factors such as arousal or general attention. Results showing that error of saccade endpoints were greater for memory-guided saccades than visually guided saccades (Figures 4B,C) were also consistent with a previous study of FEF inactivation (Dias and Segraves, 1999). In addition to contraversive saccade deficits, we also found changes in some parameters of ipsiversive saccades in monkeys *R* and *N*. These deficits might result from the mass effect of brain edema that pushed the other side of the brain (Figure 2, monkey *R*), or might be due to the large extent of FEF lesions (Peel et al., 2014). In addition to deficits in saccades, we also found bidirectional reduction of smooth pursuit velocity (Figure 5), which was also consistent with previous studies (Keating, 1991; Shi et al., 1998).

Our data of MR images and histological sections showed that radiosurgical lesions were not confined to the FEF, but extended anteriorly to the prefrontal cortex. Consistent with this, the proportion of error trials with early saccades toward the visual cue increased in the memory-guided saccade trials (Figure 4C, bottom row). This was likely because the task required suppression of unnecessary saccades (Guitton et al., 1985; Condy et al., 2007) and spatial working memory (Funahashi et al., 1989), both of which are processed in the dorsolateral prefrontal cortex. Furthermore, we found impaired contralateral limb movements in two monkeys approximately 2 months following irradiation. This could be because the necrosis surrounded by edema also posteriorly invaded the premotor and even primary motor cortices (Figure 3B). Thus, the effects of radiation unexpectedly extended beyond the FEF. In this first attempt to apply radiosurgical techniques to neuroscience research, we used 130–150 Gy to induce clear behavioral deficits. However, in future studies, we should definitely choose much lower doses for creating discrete lesions, especially within the subcortical structures. We also need to take more care of dose distribution and to investigate the precise relationship between absorbed dose, MR imaging, and pathological findings.

A notable finding in the present study was that initial changes in the MR images were dissociated from the time courses of oculomotor deficits in at least two out of three monkeys. In accordance with the appearance of brain edema, eye movements were impaired within several weeks after irradiation, but recovered in a few months (Figure 4C, monkeys *R* and *C*). These results indicated that neurons exhibited a temporary loss of function in parallel with the early development of edema in white matter, but they resumed their function likely because the edema did not entirely remove neurons. However, the MR images consistently showed signs of edema even during functional recovery (Figure 2), suggesting that chronic edema may not necessarily accompany neuronal dysfunction. Thereafter, over the 3 months following irradiation, oculomotor performance again deteriorated (Figures 4, 5), possibly because neurons in the FEF gradually lost function during the course of development. Thus, our results suggest that dysfunction of the FEF following irradiation might reflect two pathological changes: early edema in the white matter and the late necrosis in the surrounding gray matter.

### Perspective of the Primate Model of Radiosurgical Brain Lesions

The effects of irradiation on normal brain tissues and their impacts on brain function remain elusive, because only a few studies have performed the long-term follow-up of irradiation in experimental animals (Young et al., 1998; Niranjan et al., 2004). Our data suggest that, in the primate brain, the tissue damage causing irrecoverable behavioral changes might occur within 3 months of radiosurgery. Unfortunately, however, the variable timing of behavioral changes between animals made it difficult to estimate the exact timing of pathological changes after irradiation. Therefore, the aid of MR images appears to be necessary for experiments using radiosurgical techniques.

Despite a variety of techniques for establishing specific brain lesions in experimental animals, surgical ablation remains advantageous for generating relatively large, circumscribed brain lesions (Mansouri et al., 2007; Takaura et al., 2011). For example, electric coagulation and injection of neurotoxins are suitable for creating discrete lesions with a pinpoint accuracy, but these lesions are limited to a small area. While ligation of a feeding artery and cooling of brain tissue are useful for creating large lesions, it is generally difficult to control the size of the affective area. Compared with these existing techniques, radiosurgery has several promising features: (1) it is a non-invasive technique with no need for craniotomy or surgical skills; (2) it can be performed in a short period of time; and (3) it is technically possible to create subcortical lesions without damage to the cerebral cortex. Because of these features, radiosurgical techniques are now clinically applied to treat brain tumors and vascular malformations (Shaw et al., 2000; Greene-Schloesser et al., 2012), as well as for functional neurosurgery that targets subcortical structures (Ohye et al., 2012; Kooshkabadi et al., 2013). Although the radiosurgical dose used in this study generated significant edema resulting in necrotic lesions with an unexpected size (Figure 3B), a lower dose could reduce these acute changes and might be suitable for the generation of focal brain lesions. In fact, previous studies showed that the size of necrotic lesions depended on a radiation dose in the range of 25–200 Gy (Kondziolka et al., 1992; Ishikawa et al., 1999). Exploration of suitable parameters is an important topic in future studies.

As outlined in the Introduction, the radiosurgical techniques can be applied to psychophysical assessment of brain functions in experimental animals. Given the similarity of brain architectures and behavioral modalities to humans, such analyses would be more beneficial if non-human primate models were available. In fact, previous studies successfully replicated cognitive tasks in monkeys with brain lesions and clarified the causal roles of cortical areas (Passingham, 1975; Petrides, 1995; Buckley et al., 2009). Conversely, the difference in neurophysiological functions and metabolic pathways makes it difficult to replicate neurological symptoms in rodents (Blesa et al., 2012; Porras et al., 2012). In addition to the analysis of localized neural functions, the primate model of brain lesions could also be used to test therapeutic treatments of neurological disorders and to explore the mechanisms of functional recovery (Lawrence and Kuypers, 1968; Nishimura et al., 2007). Because the motor dysfunction following irradiation was relatively stable for several months (Figures 4, 5), it might be possible to use a primate model to test pharmacological or neurosurgical treatments that could be applied to patients with specific brain lesions.

Radiosurgical techniques for establishing discrete subcortical lesions in the primate brain may also directly apply to functional radiosurgery for patients with neurological and psychiatric disorders. At present, functional neurosurgery may remove epileptic tissues, electrically coagulate discrete brain regions, or stimulate specific pathways or centers through chronic electrodes. However, these treatments require invasive procedures and neurosurgical skills. Recent developments of radiosurgical techniques make it possible to ablate deep brain structures that are the targets of functional neurosurgical treatments in a less invasive manner (Young et al., 1998; Kondziolka et al., 2011; Ohye et al., 2012). The non-human primate model of radiosurgery might be useful for further developing these techniques and to assess the long-term pathological changes in normal brain tissues following irradiation.

## Conflict of Interest Statement

The authors declare that the research was conducted in the absence of any commercial or financial relationships that could be construed as a potential conflict of interest.
